# Weed germinable seedbanks of rice–wheat systems in the Eastern Indo‐Gangetic Plains: Do tillage and edaphic factors explain community variation?

**DOI:** 10.1111/wre.12505

**Published:** 2021-09-23

**Authors:** Carolyn J. Lowry, Daniel C. Brainard, Virender Kumar, Richard G. Smith, Madhulika Singh, Pankaj Kumar, Ajay Kumar, Vipin Kumar, Rajiv K. Joon, Raj K. Jat, Shishpal Poonia, Ram K. Malik, Andrew McDonald

**Affiliations:** ^1^ Plant Science Department Pennsylvania State University University Park PA USA; ^2^ Department of Horticulture Michigan State University East Lansing MI USA; ^3^ International Rice Research Institute (IRRI) Los Baños Philippines; ^4^ Department of Natural Resources and the Environment University of New Hampshire Durham NH USA; ^5^ International Maize and Wheat Improvement Center (CIMMYT)‐CSISA Hub Patna India; ^6^ CIMMYT‐India New Delhi India; ^7^ Borlaug Institute for South Asia Pusa Samastipur India; ^8^ CIMMYT South Asia Regional office Kathmandu Nepal; ^9^ School of Integrative Plant Science Cornell University Ithaca NY USA

**Keywords:** conservation tillage, rice, weed community composition, weed diversity, wheat, zero‐tillage

## Abstract

Zero tillage (ZT) is widely promoted throughout India's Eastern Indo‐Gangetic Plains (IGP) because of its potential to increase wheat productivity and resilience to abiotic stresses. Weeds remain a major barrier to ZT adoption, yet it remains unclear how ZT will influence weed communities in the Eastern‐IGP. The primary objective of this study was to characterise the composition of the germinable weed seedbank sampled just prior to the wheat phase of rice–wheat farms in Bihar and Eastern Uttar Pradesh, and examine whether adoption of ZT wheat has shifted weed community composition compared to conventional tillage (CT). Additionally, we examined whether edaphic properties and topography (upland vs. lowland) explain variation in germinable weed seedbank communities. In December 2014, we evaluated the germinable seedbank from 72 fields differing in their historic (>=3 year) tillage practices (ZT vs. CT) in three regions: Samastipur–Vaishali–Muzaffarpur (SVM), Ara–Buxar and Maharajgunj–Kushinagar. Weed community composition and species richness varied by region and topography. ZT adoption was associated with lower relative density of *Chenopodium album* in the germinable seedbank and lower emergence of *Phalaris minor* seedlings within farmers’ fields. In upland topographies of the SVM region, ZT adoption was also associated with greater relative abundance of *Solanum nigrum* in the weed seedbank. However, differences between tillage systems in individual species were not large enough to result in detection of differences at the whole‐community level. Variation in edaphic properties, most notably soil texture and pH, explained 51% of the variation in the weed seedbank community. Our work suggests several frequent but poorly understood species (e.g. *Mazus pumilus* and *Grangea maderaspatana*) in Eastern IGP for which future research should quantify their effects on crop yields. Finally, future work surveying weed species abundance at harvest could further determine the dominant problematic species in these regions.

## 1 INTRODUCTION

Wheat and rice are the dominant staple crops within India's Indo‐Gangetic Plains (IGP), with both cereals rotated within a single year (Chauhan et al., 2012). Since the 1960s, wheat yields in India have increased 280% (FAO, 2019). However, wheat yield within the Eastern IGP states (including Bihar, West Bengal and the eastern part of Uttar Pradesh) lag behind wheat yield found in the Northwestern IGP (Haryana, Punjab and western Uttar Pradesh; FAO, 2019; Paulsen et al., 2012), despite the Eastern IGP having greater access to natural resources (e.g. land and groundwater). For example, in 2016–2017, the average wheat yield in Bihar (2.43 t/ha) was approximately 50% lower than Northwestern IGP states of Punjab and Haryana (MoA, 2017).

Terminal heat stress during grain‐fill is one of the major causes of low wheat yield in the Eastern IGP (Jain et al., 2017). Planting late exacerbates the risk of terminal heat stress of wheat, and within the Eastern IGP, it is estimated that everyday wheat planting is delayed beyond mid‐November results in a yield reduction of 27.6 kg/ha (Tripathi et al., 2005). Based on a recent survey in Bihar and eastern Uttar Pradesh in 2018, 82% of farmers planted their wheat beyond the optimal planting window (Malik et al., 2019). Traditionally farmers utilised multiple tillage passes and planking to create a friable seedbed, which greatly contributed to the long turnaround period between rice harvest and wheat planting. Eliminating tillage prior to wheat planting (referred to as zero‐tillage) reduces the time required for land preparation, thereby enabling earlier planting, which ultimately reduces the risk of terminal heat stress and increase wheat productivity (Kumar et al., 2018).

Zero‐tillage (ZT) is widely promoted throughout the IGP of India because of its potential to increase wheat productivity, profitability and resource use efficiency (Erenstein and Laxmi, 2008). ZT has been widely adopted in India's Western IGP (Erenstein and Laxmi, 2008), but ZT adoption has been lower in the Eastern IGP due, in part, to limited education and extension efforts (Aryal et al., 2018), as well as a limited number of service providers that own a grain drill capable of planting wheat in untilled soil (Erenstein and Laxmi, 2008). With increased research and investment in India's Eastern IGP, the number of ZT service providers is expanding. For example, in Bihar, the number of service providers has increased from 17 in 2011 to over 1,600 by 2014 (Keil et al., 2017). The increased service providers have enabled greater ZT adoption, which in Bihar has resulted in an additional yield gain of 0.5 t/ha (19%) and economic gain of US$ 110 ha^−1^ (Keil et al., 2017).

Weeds are a major factor limiting yields within wheat production in the IGP, and are also a major barrier to adoption of ZT practices (Kumar et al., 2013). Tillage is the dominant form of weed control in rice–wheat systems, especially in the Eastern IGP which has limited access to herbicides. For example, a recent survey in Bihar found that only 49% of farmers apply herbicides, while 31% farmers do not use any weed management tactics in wheat (Kumar et al., 2019). In contrast to the arid Western region of India's IGP, the relatively humid Eastern IGP contains different, and often a more diverse, weed community (R.K. Malik, personal communication). However, to our knowledge, no studies have characterised the dominant weeds in the Eastern IGP, or the potential impact of ZT adoption on weed communities in this region.

Factors other than tillage may influence weed community composition in the Eastern IGP, such as edaphic properties and topography. In other regions, edaphic properties, such as soil texture, pH and nutrient status, have been shown to be strong predictors of the variation in weed communities (Fried et al., 2008; Korres et al., 2017; Smith et al., 2018). Rice–wheat production in the Eastern IGP occurs in both upland (less flooding and drier) and lowland (more flooding and wetter) topographies, which likely cause variation in weed community composition due to variation in flooding and soil drainage. For example, lowland rice systems in Java have greater rice weed diversity compared to upland ecosystems (Kumalasari and Bergmeier, 2014); however, to our knowledge, there has been no work characterising differences in wheat weed communities within farms located in either an upland versus lowland topography.

The composition of the germinable seedbank determines which weed species, especially annuals, could potentially infest the succeeding crop (Mahé et al., 2020; Smith et al., 2018). Additionally, the composition of the germinable seedbank reflects the success of weed management in proceeding years, because relative competitive success and fecundity will influence relative abundance in the seedbank of subsequent years. Seed longevity of many weed species within the soil seedbank can serve as a reservoir for annual weed infestations (Bàrberi and Lo, 2001; Fracchiolla et al., 2018), thereby buffering against short‐term changes to agricultural management. Additionally, examining the weed seedbank allows the examination of the potential weed community without the influence of current agronomic practices.

Therefore, the primary objective of this study was to characterise the community composition of the germinable weed seedbank collected just prior to the wheat phase of rice–wheat farms in Bihar and Eastern Uttar Pradesh. We were especially interested in whether the overall weed community of the germinable seedbank as well as the prevalence of key problematic weed species (most notably, *Phalaris minor*) differed among farms that adopted ZT compared to conventional tillage (CT). An additional objective of our study was to examine to what extent variation in edaphic properties explain variation in the weed seedbank communities. Finally, secondary objectives included: (a) to determine whether populations of key weed species emerging in situ within farmers’ fields differed between farms using ZT versus CT, and (b) examine whether land topography (upland and lowland) account for variation in the weed seedbank communities in this region.

## 2 MATERIALS AND METHODS

### 2.1 Study region and seedbank sampling

**FIGURE 1:**
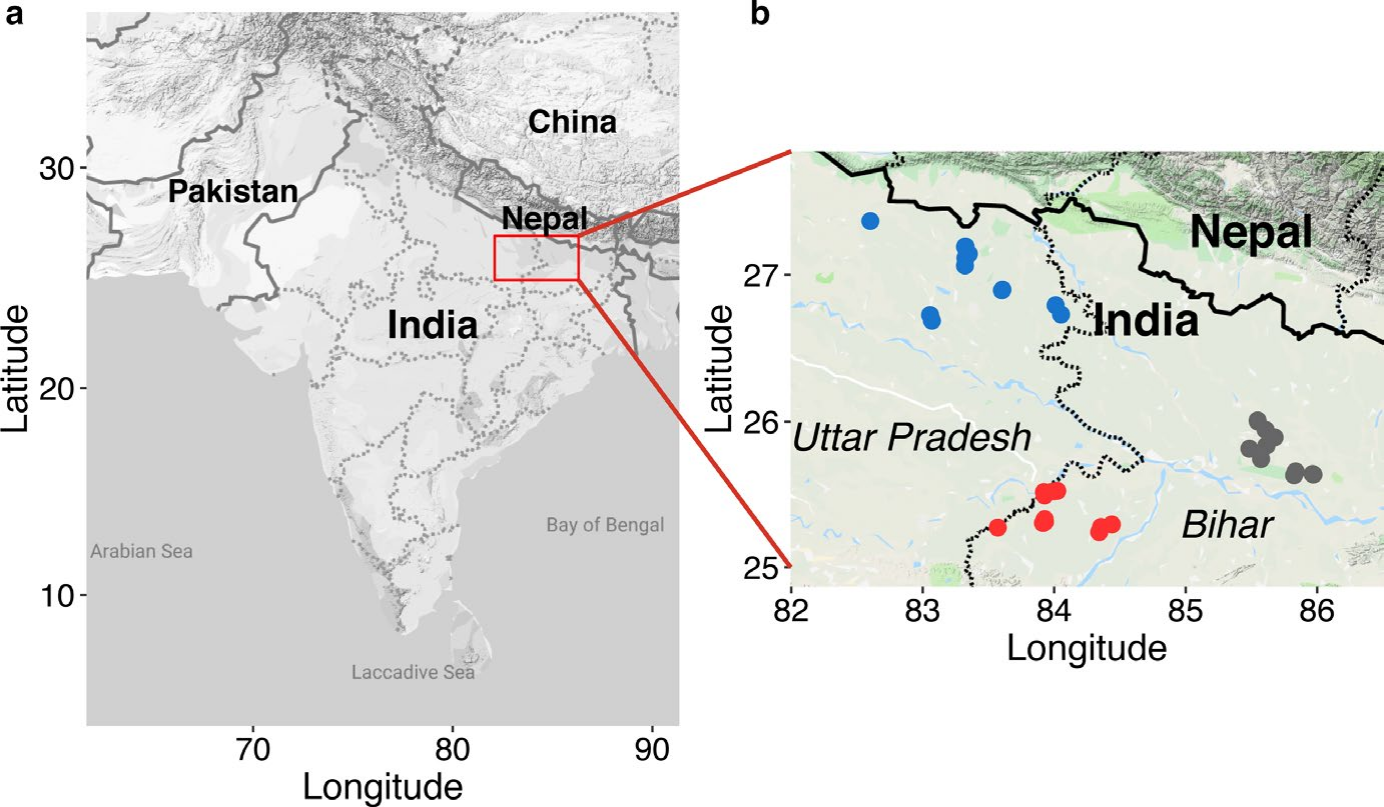
Map showing (a) region within India where seedbank sampling occurred; and (b) locations of the 72 farmers’ fields sampled for the survey of weed seedbank communities in three regions of Bihar and Eastern Uttar Pradesh: SVM (grey), AB (red) and MK (blue)

We collected soil samples for weed seedbank assessment from 72 farm fields distributed over three regions in the Eastern IGP (Figure 1): Samastipur–Vaishali–Muzaffarpur (SVM), Ara–Buxar (AB) in Bihar and Maharajgunj–Kushinagar (MK) in Eastern Uttar Pradesh. The field sites were within the project domain area of the Cereal Systems Initiative for South Asia (CSISA; www.csisa.org) in which CSISA is promoting ZT wheat. Therefore, it was only in these regions that we were able to find pairs of neighbouring fields with similar soils and microclimates but differing in their tillage history. All fields sampled were in rice–wheat grown within an annual rotation, with rice grown during the warmer rainy (*Kharif*) season, and wheat grown during the cold and drier (*Rabi*) season. All ZT farms had been utilising ZT prior to wheat for between 3 and 4 years prior to sampling, and prior to that they had been utilising CT. The CT farms had been practicing CT for over 4 years. Tillage only varied prior to wheat planting, and the sampled farms both relied on intensive tillage prior to rice planting. All the farm sites within the MK and AB regions were located in lowland topography (historically relied on flooding for rice production), while farm sites in the SVM region spanned both upland and lowland topographies. In total, 36 pairs of CT and ZT fields (six in AB, 15 in MK and 15 in SVM) ranging from 0.1 to 2 ha were sampled across all three regions.

Soil samples were collected from November 2014 to December 2014, following rice harvest but prior to wheat planting. Within each field, we collected 13 soil cores (4 cm diameter, 13 cm depth using a soil auger to approximate the depth of the tillage zone), at least 3 m from the field edges, in a W‐shaped transect with approximately 3 m between each core (see Figure S1 for sampling design). The 13 soil cores were combined and homogenised by hand, then air‐dried to prevent germination prior to the greenhouse germination assay. These air‐dried soil samples were split so that half would be sent for soil texture and chemical analysis and half used for greenhouse germination assay. Additionally, at each of the farms, we collected two bulk density cores using a bulk density corer (5 cm diameter and 12 cm depth). This soil was dried then weighed to obtain mass of soil per unit volume.

We submitted approximately 1 L total volume of soil to the Soil Test Services, TATA Chemicals Limited (Aligarh, India) for soil chemical analyses, which included soil pH, electrical conductivity (EC), organic carbon (OC), P, K, Mg, S, B, Cu, Fe and Ca. A separate subsample was utilised for soil texture analysis.

### 2.2 Greenhouse assay for germinable seedbank

In December 2014, we brought soils to a common passively heated greenhouse at the Borlaug Institute for South Asia in Pusa, Samastipur, Bihar, and evaluated the germinable seedbank through exhaustive germination. In brief, we filled greenhouse flats (25 × 35 × 5 cm) to a depth of approximately 3 cm with potting soil (3:2:1 perlite, peat and vermiculite). We then covered the layer of potting soil with fine (‘noseeum’) mesh to ensure the field soil containing weed seeds did not mix with the bottom layer. On top of the mesh, we spread 500 ml of field soil (collected from the farmers’ fields), which was mixed with perlite and vermiculite in 2:1:1 ratio to minimise differences in soil moisture retention across soil types. We prepared two flats (each with 500 ml of field soil) for a total of 1 L of soil per field. Once assembled, each flat was irrigated with subsurface irrigation as needed. Our methods (using a standard volume of homogenised soil within the seedbank flats) prevent us from precisely extrapolating species densities to an area basis; however, they provide a reliable assessment of relative differences in species abundance. Therefore, for each farm field, we calculated relative density (RD) by dividing the number of individuals of a target species by the number of individuals of all the species, and then multiplying by 100.

All weed seedlings that emerged from the flats were identified, counted and removed regularly (approximately every 3–7 days) over the duration of the germination assay. Species that could not be immediately identified were carefully transplanted into pots to allow further growth until identification was possible. When weed emergence ceased (after approximately 2 months), the weed seedbank flats were dried, stirred and then re‐watered to stimulate additional emergence for another 2 months, after which germination assays were terminated due to limiting resources. Because most of our germinable seedbank assay occurred in a passively heated greenhouse during the cooler *Rabi* season, it is likely that the weeds found in our study are more indicative of weeds commonly found during the wheat phase of the rice–wheat rotation, and weeds generally found in rice are likely underrepresented.

### 2.3 In‐field weed seedling density

We recorded weed seedling density in five randomly placed 0.25 m^2^ quadrats per field just prior to first post‐emergent herbicide application approximately 1 month after wheat planting (no pre‐emergent herbicides were used). Due to difficulty in identifying more rare species at the seedling stage, we limited seedling emergence counts to those weed species thought to be most common and troublesome (species included *Phalaris minor*, *Solanum nigrum*, *Chenopodium album*, *Lathyrus aphaca*, *Anagallis arvensis*, *Fumaria indica* and *Rumex dentatus*). Additionally, due to limited time and resources, and difficulty in reaching sites prior to herbicide applications, weeds were only counted in the AB and MK regions.

### 2.4 Data analysis

To characterise the germinable weed seedbank communities within each field, we totalled the counts of all emerged weeds for each species from both flats for each field sample (total of 1 L of soil). We then calculated the RD of all species that germinated and species richness per field (the sum of all species represented within a sample).

Linear, mixed‐effect models were used to evaluate the effects of tillage history and region on variation in weed species RD, seedbank community diversity and edaphic properties using the *nlme* package in R (Pinheiro et al., 2018). Fixed factors included tillage, region and the interaction between tillage and region. To increase normality, all species RD data were arcsine transformed and all the edaphic data (EC, OC, P, K, Mg, S, B, Cu, Fe, Ca, bulk density, soil texture) except pH were log transformed. A block effect consisted of each adjacent pair of CT and ZT farm fields and was included as a random factor within the model. To control for the inflated potential of falsely rejected null hypotheses when analysing species RD or edaphic variables, we applied the false discovery rate (Benjamini and Hochberg, 1995) to adjust *p* values by the number of weed species examined (*n* = 10, because we only examined the 10 most frequently found species) or the number of soil variables (*n* = 17). Statistical differences were considered significant when α < 0.05.

To characterise variation in weed seedbank community composition and to evaluate whether this variation could be explained by tillage or edaphic properties, we conducted a series of multivariate analyses. All multivariate analyses were performed using a Bray–Curtis dissimilarity matrix created from species RD data, which were fourth‐root transformed to decrease the influence of abundant species (Reberg‐Horton et al., 2006). To reduce the influence of rare species on the community analyses, we removed all species that were present at less than 5% of fields.

We used non‐metric multidimensional scaling (NMDS) ordination to characterise variation in germinable weed seedbank community composition (McCune and Grace, 2002). NMDS was performed using the *metaMDS* function in the vegan package in R (Oksanen et al., 2019) with 250 runs of the ordination. The ideal number of dimensions was selected when additional dimensions no longer reduced stress by a unit greater than five, and a Monte Carlo test using randomised data confirmed whether the NMDS dimension reduction was stronger than would be predicted by chance (McCune and Grace, 2002). To evaluate whether weed communities differed based on region or tillage history, we performed permutational multivariate analysis of variance (PERMANOVA) based on 999 permutations using the *adonis* function in the vegan package in R. Finally, to examine which species were responsible for driving differences identified by the PERMANOVA, we performed an indicator species analysis using the *multipatt* function in the indicspecies package in R (Caceres and Legendre, 2009).

To examine how variation in weed community composition related to site edaphic properties, we performed a partial least‐squares regression (PLSR) using the *plsr* function in the pls package in R (Bjørn‐Helge et al., 2019). The predictive matrix included all edaphic variables (pH, EC, OC, P, K, Mg, S, B, Cu, Fe, Ca, bulk density, soil texture). The response variables were the individual NMDS ordination axis scores. Predictor variables with variable importance values greater than 0.8 were retained in the final models (Smith et al., 2018), and model predictive ability was determined with *K*‐fold cross‐validation (*K* = 10).

To examine whether the weed seedbank community varied by land topography (upland vs. lowland), and whether topography interacted with tillage or edaphic properties, we performed an NMDS, PERMANOVA, indicator species analysis and PLSR all as described above. However, because all farm sites within both the MK and AB regions were exclusively located in lowland topography, analyses examining variation in seedbank weed communities due to topography were exclusively performed on soil seedbanks from farm sites within the SVM region.

## 3 RESULTS

### 3.1 Tillage history and regional differences in species diversity and weed community composition

We found a total of 32 species from 16 different plant families across the three sampling regions in Bihar and Eastern Uttar Pradesh (Table 1). Species richness varied by region and by tillage history. Mean species richness ranged from approximately eight species in AB to approximately ten species in MK and SVM (*F*_2, 33_ = 4.1; *p* = 0.026). Farms that adopted ZT had one more species compared to those using CT (*F*_1, 33_ = 4.4; *p* = 0.044).

**TABLE 1:** The relative frequency (% presence in sampled fields) of all weed species found in the germinable weed seedbank from farmers’ fields sampled just prior to wheat planting

Latin name^a^	Common name^a^	%
*Grangea maderaspatana* L. Poir	Madras carpet	88.9
*Chenopodium album* L.	Common lambsquarters	77.8
*Polygonum plebeium* R. Br.	Common knotweed	70.8
*Mazus pumilus* (Burm. f.) Steenis	Japanese mazus	63.9
*Polypogon monspeliensis* (L.) Desf.	Rabbitfoot polypogon	62.5
*Digitaria ciliaris* ^b^ (Retz.) Koeler	Southern crabgrass	50
*Anagallis arvensis* L.	Scarlet pimpernel	48.6
*Phalaris minor* Retz.	Littleseed canarygrass	40.3
*Solanum nigrum* L.	Black nightshade	36.1
*Digitaria* sp.		34.7
*Pouzolzia zeylanica* (L.) Benn.	Graceful Pouzolz's Bush	27.8
*Soliva anthemifolia* (Juss.) Sweet	Button burweed	23.6
*Eclipta prostrata* (L.) L.^b^	False daisy	20.8
*Amaranthus spinosus* L.	Spiny amaranth	13.9
*Oxalis corniculata* L.	Creeping woodsorrel	12.5
*Parthenium hysterophorus* L.	Carrot grass	12.5
*Rumex dentatus* L.	Toothed dock	12.5
*Gnaphalium* sp.		11.1
*Melilotus indica* (L.) All.	Indian sweetclover	11.1
*Physalis minima* L.	Native gooseberry	11.1
*Cucurbit* sp.		9.7
*Portulaca quadrifida* L.	Chicken weed	9.7
*Alternanthera sessilis* (L.) R.Br. ex DC.^b^	Sessile joyweed	6.9
*Caesulia axillaris* Roxb.^b^	Pink Node Flower	6.9
*Cannabis sativa* subsp. indica (Lam.)	Marijuana	4.2
*Euphorbia thymifolia* L.	Gulf Sandmat	4.2
*Lathyrus aphaca* L.	Yellow pea	4.2
*Medicago polymorpha* var. *denticulata* (Willd.) Kerguélen	Burr medic	4.2
*Vicia sativa* L.	Common vetch	4.2
*Brassica juncea* (L.) Czern.	Mustard	2.8
*Eleusine indica* (L.) Gaertn.	Indian goosegrass	2.8
*Fumaria indica* (Hausskn.) Pugsley	Indian fumitory	2.8
	*Number of sites*	72

^a^ Flowers of India, 2018.

^b^ *Digitaria ciliaris*, and *Eclipta prostrata*, *Alternanthera sessilis* and *Caesulia axillaris*, are typically thought to be weeds in the rice phase of crop rotations in the region.

**FIGURE 2:**
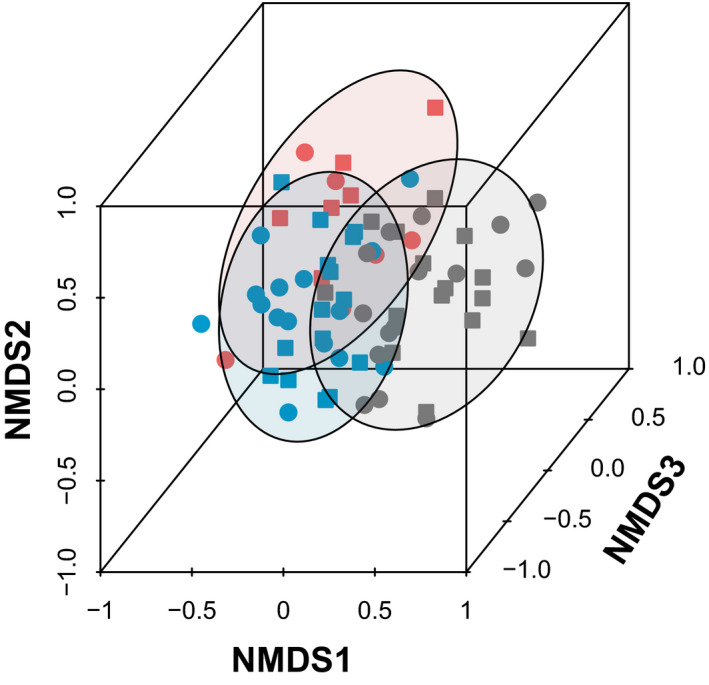
Plot scores for the three‐dimensional solution of the NMDS ordination performed on the RD of weed species within the soil germinable seedbank of farmers’ fields in three regions of Bihar and Eastern Uttar Pradesh: SVM (grey), AB (red) and MK (blue). NMDS: stress = 18, *p* < 0.05, cumulative *R* ^2^ = 74.8%. Farm sites utilised either conventional tillage (CT, circle) or squares (ZT, squares). Ellipses represent 90% confidence interval

The NMDS ordination resulted in a three‐dimensional solution that accounted for 73% of the variation of the original distance matrix and indicated weed germinable seedbank communities varied by region but not by tillage (Figure 2). PERMANOVA confirmed the community varied by region (PERMANOVA *F*_2, 66_ = 6.8, *p* = 0.001) but indicated no effect of tillage history (PERMANOVA *F*_1,66_ = 1.4, *p* = 0.18) and no region by tillage history interaction (PERMANOVA *F*_2, 66_ = 0.79, *p* = 0 0.75).

**FIGURE 3:**
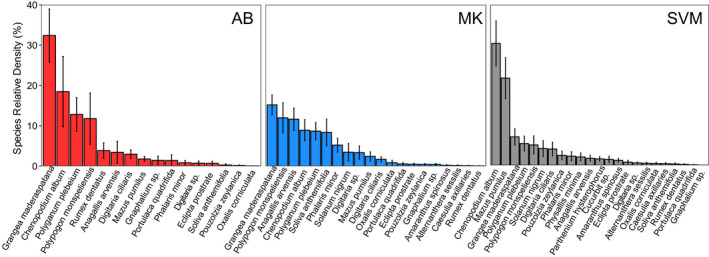
Mean (± standard error of the mean [SEM]) RD (percent of all individuals of a particular species) of weed species that emerged from the germinable soil seedbank from farmers’ fields in AB (red and left), MK (blue and centre) and SVM (grey and right)

According to indicator species analyses, species found to be indicative of the AB region (*p* < 0.05) included *Rumex dentatus* (IndVal = 0.66), *Grangea maderaspatana* (IndVal = 0.65) and *Polygonum plebeium* (IndVal = 0.63); species indicative of the MK region included *Anagallis arvensis* (IndVal = 0.67), *Phalaris minor* (IndVal = 0.60) and *Soliva anthemifolia* (IndVal = 0.61) and finally, species indicative of the SVM region (*p* > 0.05) included *Mazus pumilus* (IndVal = 0.67), *Chenopodium album* (IndVal = 0.66), *Solanum nigrum* (IndVal = 0.56), *Parthenium hysterophorus* (IndVal = 0.55), *Physalis minima* (IndVal = 0.52) and *Cucurbit* sp. (IndVal = 0.46). These regional differences in the weed community in the germinable seedbank were largely reflected in the regional variation in the mean RD of species by region (Figure 3). Species that were relatively common but not unique to any of the three regions included *Amaranthus spinosus*, *Polypogon monspeliensis* and *Oxalis corniculata*.

**FIGURE 4:**
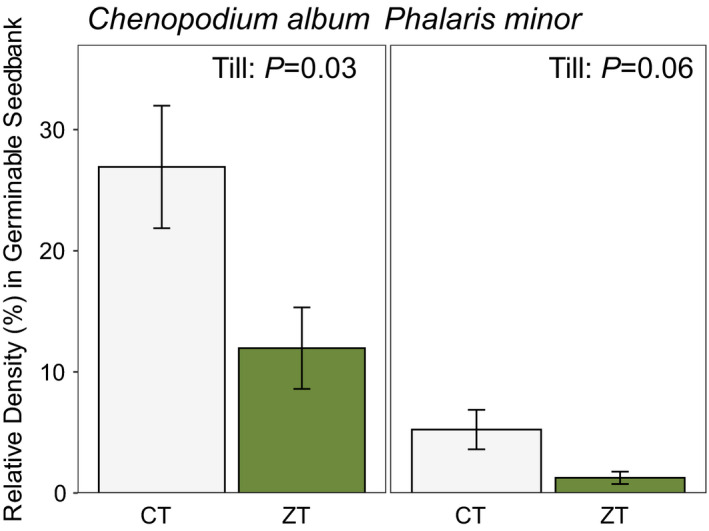
Mean RD (± SEM) of *Chenopodium album* (left) and *Phalaris minor* (right) seedlings emerged within germinable soil seedbank from soil collected from farmer's fields in both conventional (CT) and zero‐till (ZT) rice–wheat farms in the Eastern IGP just prior to the wheat phase of the rotation

While the weed seedbank community did not differ between the two tillage systems (see results from PERMANOVA above), we did find lower RD of *Chenopodium album* (*F*_1, 52_ = 8.2; *p* = 0.03), as well as a trend towards lower RD of *Phalaris minor* (*F*_1, 52_ = 6.7; *p* = 0.06), within the ZT compared to the CT fields (Figure 4). However, the RD of other weed species did not differ by tillage history (for all other species, *p* > 0.05, see Table S1). While our methods (using a standard volume of homogenised soil within the seedbank flats) prevent us from precisely extrapolating species densities to an area basis, they provide a reliable assessment of species RD. Moreover, we found no difference in bulk density between tillage systems as mean bulk density in both tillage systems was 1.5 g/cm^3^, (CT SE = 0.015, ZT SE = 0.016); therefore, observed effects of tillage on weed RD imply differences on a per area basis as well.

**FIGURE 5:**
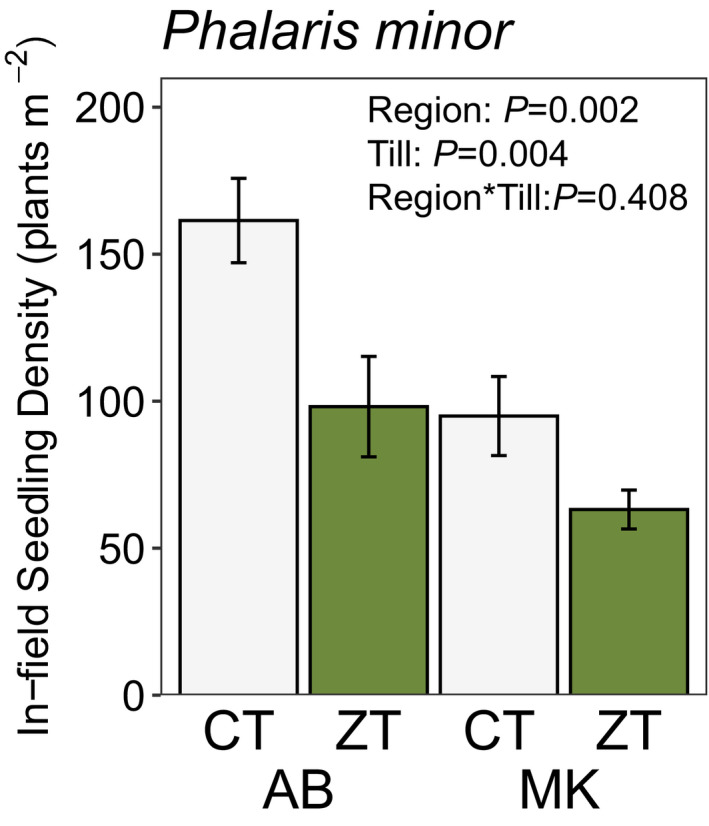
Mean (± SEM) density of *Phalaris minor* seedlings emerged within farmers’ fields in both conventional (CT) and zero‐till (ZT) rice–wheat farms in the AB and MK regions of the Eastern IGP

We also found lower seedling density of *Phalaris minor* emerged within farmers’ fields during wheat growth in ZT versus CT fields in the AB and MVK regions (*F*_1,30_ = 12.1; *p* = 0.004, Figure 5). However, we found no other differences in the density of weed species that emerged in farmers’ fields varying in tillage history (Table S2).

### 3.2 Tillage and regional variation in edaphic properties

We found considerable variation in edaphic properties among the three regions sampled within our study (Table 2). For example, the AB region was characterised by clay loam soils, with approximately double and triple the percent clay of the MK and SVM regions (*F*_2, 35_ = 41.7, *p* < 0.001) respectively. Soils within the SVM region were primarily silty loams, while the soils within the MK region were mostly medium or sandy loam soils, with the highest sand content (*F*_2, 35_ = 5.0, *p* = 0.03). Soils within all three regions were relatively basic (pH ranged from 6.9 to 8.64). Unsurprisingly, the three regions also varied in those soil cation micro‐nutrients which tend to co‐vary with soil pH levels, including Mn, Fe and Cu (*p* < 0.05). Soils from ZT (1.3 ppm) fields had an approximately 40% higher concentration of Zn compared to CT (0.9 ppm, *p* = 0.03). However, we found no relationship between tillage history and the other edaphic properties (*p* > 0.05).

**TABLE 2:** Mean edaphic variables for each of the three regions sampled for the seedbank survey

Edaphic Variables	AB	MK	SVM	*p* value*
Silt (%)	28.9b	31.3b	52.1a	<0.001
Clay (%)	29.7a	16.5b	8.4c	<0.001
Sand (%)	41.5b	52.2a	39.6b	0.030
Bulk density (g/cm^3^)	1.6a	1.5b	1.5b	0.04
pH	7.7 b	7.4c	8.3a	<0.001
EC (ds/m)	0.78a	0.41b	0.78a	<0.001
Organic C (%)	0.63	0.71	0.71	1.00
P (kg/ha)	74.5	78.1	92.8	0.82
K (kg ha)	62.6	36.5	56.7	1.00
S (mg/kg)	22.4	10.5	20.4	0.39
B (mg/kg)	0.55	0.41	0.5	0.18
Cu (mg/kg)	2.4a	1.9b	2.5a	0.01
Fe (mg/kg)	35.1b	52.5a	19.1c	<0.001
Mn (mg/kg)	13.7a	12.4a	4.5b	<0.001
Zn (mg/kg)	1.03	1.63	0.64	0.09
Ca (mg/kg)	145a	102b	156a	0.01
Mg (mg/kg)	22.9a	12.7b	15.3b	0.02

Note

Regions include AB, MK and SVM.

* *p* values are from ANOVAs examining the effect of region on edaphic variables, using a false discovery rate correction for multiple comparisons. Different letters represent differences as determined by Fisher's protected LSD (*p* ≤ 0.05).

### 3.3 Relationship between weed germinable seedbank communities and edaphic properties

**FIGURE 6:**
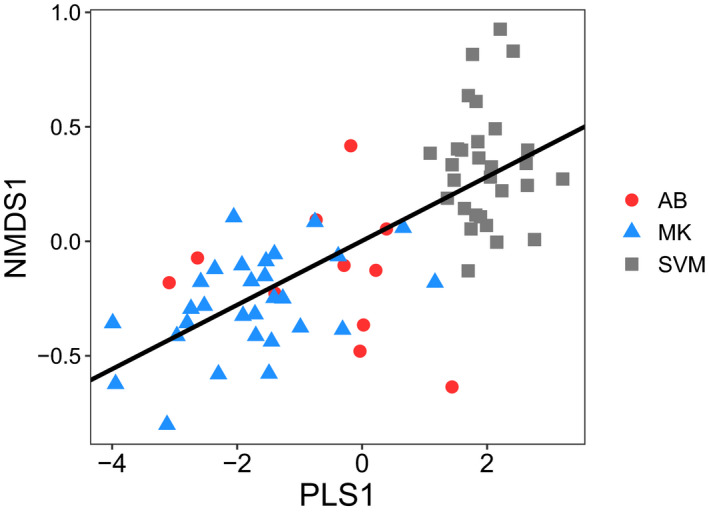
Results from partial least squares regression showing the relationship between the PLS scores comprised edaphic variables and the first axis from the NMDS of the weed seedbank community. Variables loading most strongly on the PLS1 component include pH (0.46), Mn (−0.43), Fe (−0.39), Ca (0.35), %Silt (0.34), EC (0.29), %Clay (−0.29) and Zn (−0.23). This PLSR factor accounted for 50% of the variation in the listed edaphic variables and explained approximately 51% of the variation in the first NMDS axis

PLSR was used to evaluate the degree to which variation in edaphic characteristics explained variation in the weed communities across the sites (characterised by the three NMDS ordination axes). Analysis of the first NMDS axis indicated the most parsimonious PLSR model was a single component comprised 10 variables, which together accounted for 51% of the variation in the first NMDS axis (Figure 6). Variables (and their loading scores) on the PLS component found to predict community composition within the germinable seedbank included: pH (0.46), Mn (−0.43), Fe (−0.39), Ca (0.35), %Silt (0.34), EC (0.29), %Clay (−0.29) and Zn (−0.23). Analyses of the second and third NMDS axes did not yield an explanatory PLSR model.

As expected, we generally found the species that were significant indicators of each of the three regions also tended to be correlated with the soil properties of that region (Table S3). For example, species indicative of AB (*Grangea maderaspatana*, *Rumex dentatus*) were positively correlated with greater %Clay, while species most indicative of SVM (*Chenopodium album*, *Mazus pumilus*, *Solanum nigrum*, *Parthenium hysterophorus*, *Physalis minima* and *Cucurbit* sp.) tended to be positively correlated with pH and %Silt, and many of these species were negatively correlated with the soil nutrients in which availability is reduced at higher pH, including Mn and Fe. Finally, species indicative of MK were negatively correlated with pH and EC.

### 3.4 Variation in weed species diversity and germinable seedbank community composition between upland and lowland topographies

Species richness varied between the upland and lowland topographies in the SVM region, with upland rice–wheat farms averaging 10.2 (0.9) species compared to 7.6 (0.5) in farms located within the lowland topography (*F*_1, 13_ = 8.9, *p* = 0.011). We found no difference in edaphic properties between the upland and lowland topographies in the SVM region (Table S4).

An NMDS ordination of the weed communities within the upland and lowland topographies of SVM resulted in a three‐dimensional solution that accounted for 80% of the variation of the original distance matrix (Figure S2). A PERMANOVA revealed that the germinable weed community differed between the upland and lowland topographies of the SVM region (PERMANOVA *F*_1,26_ = 2.08, *p* = 0.034). Indicator species analysis revealed two weed species significantly associated with the upland topography (*p* < 0.05), including *Mazus pumilus* (IndVal = 0.81) and *Physalis minima* (IndVal = 0.64); however, we found no weed species that were indicative of the lowland topography.

**FIGURE 7:**
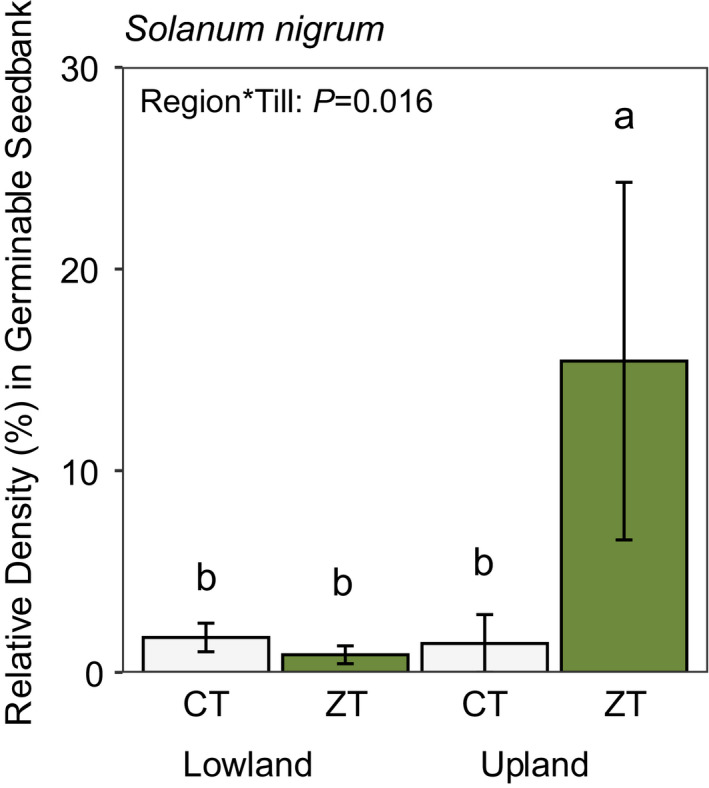
Mean RD (± SEM) of *Solanum nigrum* seedlings emerged from the germinable soil seedbank from soil collected from farmer's fields in both conventional (CT) and zero‐till (ZT) rice–wheat farms in both Upland and Lowland topographies in the SVM region in Bihar. Means followed by the same letter are not statistically different at *p* > 0.05

Mean comparisons found an interaction between tillage and topography and their effects on the RD of *Solanum nigrum* in the germinable seedbank of farmers’ fields in the SVM region (*F*_1,13_ = 15.2, *p* = 0.016). ZT was associated with roughly 900% greater *Solanum nigrum* RD compared to CT in the upland topography, but did not differ in the lowland topography (Figure 7). We found no effect of topography or tillage on the density of other weed species within the soil seedbank from farms in the SVM region (Table S5).

## 4 DISCUSSION

Our research provides a broad overview of the predominant species and community composition of germinable weeds within the soil seedbank prior to the wheat phase in farmers’ fields in Eastern Uttar Pradesh and Bihar. After 3–4 years of implementation, adoption of ZT wheat appears to have had little effect on the overall germinable weed seedbank community. We observed lower RD of *Chenopodium album* and *Phalaris minor* in the germinable seedbank from ZT compared to CT fields. We also observed greater RD of *Solanum nigrum* in ZT compared to CT in upland topographies of the SVM region. However, these differences in individual species were not large enough to contribute to the detection of tillage effects at the whole‐community level. Variation in edaphic properties, most notably soil texture and pH, explained a considerable amount of variation in the germinable weed seedbank community across the three regions sampled within this study.

### 4.1 1 Community composition did not differ between tillage systems

Our findings suggest that the transition to ZT wheat production has not led to large‐scale shifts in the community composition of the weed germinable seedbank after 3–4 years in which ZT had been adopted among farmers within the Eastern IGP of India. This is consistent with previous research showing that tillage effects on the weed seedbank are relatively slow to accumulate (Davis et al., 2005; Légère et al., 2011). For example, Légère et al. (2011) found no difference in the weed seedbank community composition between ZT, mouldboard plough or chisel plough after 5 years of tillage system implementation, but the weed community did differ after 18 years. In comparison, weed seedbank community composition did not differ after 12 years of continuous no‐till versus CT in the Midwest region of the USA (Davis et al., 2005), but did differ after continuous no‐till versus CT in Central Italy (Bàrberi and Lo, 2001). It is possible that within our study, use of ZT had occurred for too short an interval to cause any detectable effects on the germinable weed seedbank sampled prior to the wheat rotational phase. Additionally, it is possible that weed communities within CT and ZT tillage systems vary in perennial species that reproduce vegetatively, because germinable seedbank studies fail to capture these perennial species. Previous work has found higher populations of perennial species, such as bermudagrass (*Cynodon dactylon*), in ZT wheat within the IGP (Kumar et al., 2013).

Reductions in the relative seedbank density of *C. album* and *P. minor* suggested by our study are consistent with previous studies (Bàrberi and Lo, 2001; Clements et al., 1996; Shyam et al., 2014). Both species are dominant weeds of wheat grown in the IGP, and are especially problematic because of their highly competitive nature and potential reductions in wheat yields (Dodamani and Das, 2013; Singh et al., 1999).

*Phalaris minor* is one of the most problematic weeds across the IGP, and previous studies have found lower seedling emergence (Franke et al., 2007), reduced biomass and fecundity (Chhokar et al., 2007) and higher rates of post‐dispersal seed predation (Kumar et al., 2013) in ZT compared to CT. Finally, longevity of *Phalaris minor* seeds in the soil seedbank is relatively short which increases sensitivity to management (Franke et al., 2007). ZT reduction in *Phalaris minor* is especially important because this species is becoming increasingly difficult to manage due to the evolution of resistance to multiple herbicides commonly used within wheat, and in fact, one population was found to have evolved resistance to three sites of action (Heap, 2019).

### 4.2 Edaphic properties predict variation in weed seedbank community composition

Understanding the factors that shape weed communities allows us to draw inferences on how weed communities may be affected by changes in management or environmental conditions. Our findings are consistent with previous studies which have found that across relatively small geographic scales, edaphic properties can be strong predictors of weed community composition (Fried et al., 2008; Korres et al., 2017). However, other regional variation, such as differences in local crop diversity, may also have influenced the germinable seedbank community. For example, the AB region (which is almost entirely dominated by rice–wheat) has lower crop diversity than both the SVM (which includes diverse vegetables, maize and potatoes) and the MK region (which also includes sugar cane, MoA, 2017). The diversity of the local cropping systems may contribute to both the SVM and MK regions having greater weed diversity.

Within our study, soil texture and pH were the edaphic variables that explained the largest amount of variation in the weed seedbank community. Soil texture varied considerably among the three regions of our study, likely due to variation in alluvial deposits and site position within the floodplains of the Ganges, Sone, Ghaghara or Gandak Rivers. Soil texture largely influences soil water holding capacity, and along with pH, both influence the availability of soil nutrients, ultimately affecting the relative competitive ability of weed species (Weaver and Hamill, 1985). Soil texture and pH also affect seed longevity and seedling emergence, and these effects can vary by species (Hoyle et al., 2013; Pakeman et al., 2012).

The relationship between edaphic properties and community composition is especially interesting for the species which were relatively abundant in our study, but for which there is currently little information. For example, *Grangea maderaspatana* and *Mazus pumilus* were present at 88.9% and 63.9% of our fields respectively. However, neither of these species were mentioned in recent reviews characterising the predominant weeds in wheat within the IGP (Kumar et al., 2013). Several weed surveys have identified these species as present in wheat cropping systems in neighbouring regions, including Pakistan (Qureshi and Bhatti, 2001) and Nepal (Niroula, 2013), but a thorough ISI Web of Science search (July, 2019) revealed no studies that have evaluated the habitat preferences of these species, or their potential competitive effects on crops. In our study, we found *Grangea maderaspatana* to be associated with the AB region and negatively correlated with soil pH, while *Mazus pumilus* was associated with the SVM region and positively correlated with soil pH. Because of the observational nature of our study, we cannot rule out the possibility that such associations are an artefact from differences in dispersal among our three regions rather than a preference of that species or ecotype for a specific habitat. However, our results provide a baseline for testing hypotheses about key factors influencing the population dynamics of these species, including their response to edaphic properties or management practices. Future research could assess the potential adverse effects of this species on crops in the IGP; management factors influencing its abundance and appropriate alternative methods for its management.

## 5 CONCLUSIONS

This study provides a broad overview of the dominant weed species found in the soil germinable seedbank of rice–wheat farms in Bihar and Eastern Uttar Pradesh. After 3–4 years of adoption of ZT wheat, we found no difference in the overall weed community compared to CT. However, ZT adoption was associated with lower RD of *Chenopodium album* in the germinable seedbank and lower emergence of *Phalaris minor* seedlings within farmers’ fields. Both species are some of the most troublesome weeds of wheat in the IGP, suggesting that ZT can be one component of an integrated approach to managing these species. In addition, this study suggests that soil texture and pH may be important factors shaping weed communities within this region.

Future research examining the mechanistic basis for these weed–soil associations may be helpful for understanding their spread and identifying efficient management strategies. Knowledge on the relative frequency and abundance of key weed species generated through this survey will be helpful for prioritising target species for future research, especially for the frequent but poorly understood species (e.g. *Mazus pumilus* and *Grangea maderaspatana*). For these species, additional research quantifying their effect on crop yields, as well as successful control measures, may be helpful for prioritising and reducing weed management costs in these systems. Finally, future work surveying weed species abundance at harvest could further determine the dominant problematic species in these regions.

## CONFLICT OF INTEREST

The authors have no conflict of interest to declare.

## AUTHOR CONTRIBUTIONS

C.J.L., D.C.B, Virender K., R.K.M., R.K.J and A.M. conceived of the project and designed the experiment. Field work was conducted by C.J.L., M.S., P.K., A.K., Vipin K., S.P., and R.K.J. Data collection and sample processing was performed by C.J.L., M.S., P.K., A.K., Vipin K., and S.P., and R.K.J. Data was analysed by C.J.L., with input from R.G.S., D.C.B., and Virender K. C.J.L., D.C.B., and Virender K., wrote the manuscript with input from all co‐authors.

### PEER REVIEW

The peer review history for this article is available at https://publons.com/publon/10.1111/wre.12505.

## Supporting information

Supinfo S1
