# Supplementary material for: Weed germinable seedbanks of rice–wheat systems in the Eastern Indo‐Gangetic Plains: Do tillage and edaphic factors explain community variation?
Source: Weed Res. 2021 Sep 23;61(6):475–85. doi: 10.1111/wre.12505 (PMC9290468; doi:10.1111/wre.12505)
Supplement: Supplementary file 1 — Supinfo S1 [file WRE-61-475-s001.docx]

| Supplemental Table 1. Mean (and standard error) relative density of commonly found weed species in the germinable seedbank from soil collected just prior to wheat planting from conventionally tilled (CT) and zero-till (ZT) farmers' fields. | | | | | | | | | | | | | | | | | | | | | |
| --- | --- | --- | --- | --- | --- | --- | --- | --- | --- | --- | --- | --- | --- | --- | --- | --- | --- | --- | --- | --- | --- |
|  |  | *Phalaris minor* | | *Chenopodium album* | | *Anagallis arvensis* | | *Solanum nigrum* | | *Mazus pumilus* | | *Polygonum plebejum* | | *Grangea maderaspatana* | | *Polypogon monspeliensis* | | *Digitaria ciliaris* | | *Soliva anthemifolia* | |
| Region | Till | ──────────────────────────────────────────────────────── % ──────────────────────────────────────────────────── | | | | | | | | | | | | | | | | | | | |
| AB | CT | 0.8 | (0.7) | 33.6 | (15.4) | 1.4 | (1.4) | 0.0 | (0.0) | 1.1 | (0.7) | 12.0 | (6.0) | 21.5 | (9.1) | 14.0 | (11.2) | 3.6 | (2.0) | 0.0 | (0.0) |
| AB | ZT | 0.8 | (0.8) | 3.3 | (2.3) | 5.4 | (5.4) | 0.0 | (0.0) | 2.5 | (1.0) | 13.6 | (6.4) | 43.3 | (7.8) | 9.5 | (7.1) | 2.3 | (0.81) | 0.5 | (0.54) |
| MK | CT | 7.9 | (3.2) | 9.7 | (2.8) | 16.0 | (4.9) | 1.7 | (0.8) | 3.1 | (2.2) | 9.8 | (3.3) | 18.4 | (3.8) | 9.5 | (2.8) | 1.2 | (0.54) | 2.9 | (1.81) |
| MK | ZT | 2.5 | (1.1) | 8.1 | (4.5) | 7.3 | (2.3) | 5.2 | (4.3) | 1.7 | (0.4) | 7.5 | (2.8) | 12.0 | (3.1) | 14.5 | (7.0) | 2.2 | (1.13) | 13.8 | (6.09) |
| SVM | CT | 4.3 | (2.1) | 41.4 | (8.6) | 2.6 | (1.4) | 1.6 | (0.7) | 15.4 | (6.0) | 4.1 | (2.8) | 4.3 | (1.4) | 6.9 | (4.4) | 4.2 | (2.96) | 0.1 | (0.07) |
| SVM | ZT | 0.2 | (0.2) | 19.3 | (6.3) | 0.9 | (0.4) | 6.7 | (3.9) | 28.1 | (8.2) | 6.7 | (2.5) | 9.8 | (3.8) | 3.2 | (1.0) | 3.9 | (1.83) | 0.5 | (0.54) |
|  |  | Significance of Fixed Effects (P values) | | | | | | | | | | | | | | | | | | | |
| Region (R) | | 0.17 | | 0.19 | | 0.01 | | 0.62 | | 0.13 | | 0.93 | | <0.001 | | 1.00 | | 1.00 | | 0.03 | |
| Till (T) | | 0.06 | | 0.03 | | 0.78 | | 0.62 | | 0.38 | | 1.00 | | 0.92 | | 1.00 | | 1.00 | | 0.22 | |
| R*T | | 1.00 | | 0.39 | | 1.00 | | 1.00 | | 1.00 | | 1.00 | | 0.08 | | 1.00 | | 1.00 | | 0.57 | |

| Supplemental Table 2. Mean (+/- SEM) density of weed seedlings emerged within farmers’ fields in both conventional (CT) and zero-till (ZT) rice-wheat farms in the Ara-Buxar (AB) and Maharajgunj-Kushinagar (MK) region of the Eastern IGP. | | | | | | | | | | | | | | | |
| --- | --- | --- | --- | --- | --- | --- | --- | --- | --- | --- | --- | --- | --- | --- | --- |
| Region | Till | *Phalaris minor* | | *Solanum nigrum* | | *Chenopodium album* | | *Lathyrus aphaca* | | *Anagallis arvensis* | | *Fumaria indica* | | *Rumex dentatus* | |
|  |  | ─────────────────────────────────# m^-2^──────────────────────────────────── | | | | | | | | | | | | | |
| AB | CT | 161.0 | (14.4) | 2.5 | (0.8) | 33.1 | (6.7) | 36.5 | (4.5) | 0.5 | (0.5) | 0.5 | (0.4) | 2.2 | (0.5) |
| AB | ZT | 98.1 | (17.1) | 1.1 | (0.5) | 33.2 | (5.3) | 38.2 | (6.6) | 0.6 | (0.3) | 0.8 | (0.4) | 1.1 | (0.4) |
| MK | CT | 94.9 | (13.4) | 26.0 | (5.7) | 59.8 | (21.3) | 8.1 | (5.3) | 16.9 | (4.5) | 2.2 | (1.3) | 1.0 | (0.6) |
| MK | ZT | 63.1 | (6.6) | 37.2 | (6.5) | 21.4 | (3.6) | 2.7 | (1.4) | 16.6 | (3.0) | 4.2 | (2.2) | 1.0 | (0.8) |
|  | | Significance of Fixed Effects (P values) | | | | | | | | | | | | | |
| Region (R) | | 0.002 | | <0.001 | | 1.000 | | <0.001 | | <0.001 | | 0.176 | | 0.845 | |
| Till (T) | | 0.004 | | 0.310 | | 0.198 | | 0.962 | | 1.000 | | 0.837 | | 0.845 | |
| R*T | | 0.408 | | 0.310 | | 0.198 | | 0.962 | | 1.000 | | 0.998 | | 0.845 | |

| Supplemental Table 4. Mean edaphic properties measured in both upland and lowland topography in the SVM region. | | | |
| --- | --- | --- | --- |
| **Edaphic Variables** | **Lowland** | **Upland** | **P value^*^** |
| Silt (%) | 51.42 | 52.97 | 1.00 |
| Clay (%) | 9.64 | 6.58 | 1.00 |
| Sand (%) | 38.94 | 40.46 | 1.00 |
| Bulk Density (g/cm^3^) | 1.44 | 1.49 | 1.00 |
| pH | 8.28 | 8.27 | 1.00 |
| EC (ds/m) | 0.87 | 0.65 | 0.59 |
| Organic C (%) | 0.78 | 0.6 | 0.59 |
| P (kg/ha) | 97.32 | 86.05 | 1.00 |
| K (kg ha) | 55.33 | 58.67 | 1.00 |
| S (mg/kg) | 25.87 | 12.18 | 1.00 |
| B (mg/kg) | 0.5 | 0.51 | 1.00 |
| Cu (mg/kg) | 2.53 | 2.55 | 1.00 |
| Fe (mg/kg) | 17.59 | 21.33 | 0.68 |
| Mn (mg/kg) | 3.99 | 5.22 | 0.20 |
| Zn (mg/kg) | 0.65 | 0.61 | 1.00 |
| Ca (mg/kg) | 162.2 | 146.2 | 1.00 |
| Mg (mg/kg) | 17.36 | 12.29 | 0.19 |
| * P values are from ANOVAs examining the effect of topography on edaphic variables, using a false discovery rate correction for multiple comparisons. | | | |

| Supplemental Table 5. Mean relative density (+/- SEM) of weed seedlings emerged from the germinable soil seedbank from soil collected from farmer’s fields in both conventional (CT) and zero-till (ZT) rice-wheat farms in both Upland and Lowland topographies in the Samastipur-Vaishali-Muzaffarpur (SVM) region in Bihar. | | | | | | | | | | | | | | | | | | | |
| --- | --- | --- | --- | --- | --- | --- | --- | --- | --- | --- | --- | --- | --- | --- | --- | --- | --- | --- | --- |
| Ecology | Till | *Chenopodium album* | | *Mazus pumilus* | | *Grangea maderaspatana* | | *Solanum nigrum* | | *Polypogon monspeliensis* | | *Polygonum plebejum* | | *Digitaria ciliaris* | | *Phalaris minor* | | *Physalis minima* | |
|  |  | *%* | | | | | | | | | | | | | | | | | |
| Lowland | CT | 53.0 | (11.5) | 3.7 | (1.5) | 5.7 | (2.1) | 1.8 | (0.7) | 8.7 | (7.2) | 6.6 | (4.6) | 6.4 | (5.2) | 2.1 | (1.4) | 1.4 | (1.4) |
| Lowland | ZT | 25.8 | (9.2) | 21.9 | (11.3) | 13.0 | (6.1 | 0.9 | (0.5) | 3.9 | (1.6) | 10.4 | (3.9) | 5.7 | (3.0) | 0.3 | (0.3) | 0.1 | (0.1) |
| Upland | CT | 25.8 | (11.0) | 34.1 | (12.0) | 2.5 | (1.4) | 1.5 | (1.5) | 4.5 | (3.1) | 0.3 | (0.2) | 1.2 | (0.9) | 8.3 | (5.2) | 4.3 | (3.7) |
| Upland | ZT | 10.3 | (6.8) | 39.7 | (12.7) | 5.8 | (2.1) | 16.2 | (9.5) | 2.5 | (1.1) | 1.8 | (1.1) | 1.3 | (0.4) | 0.0 | (0.0) | 4.4 | (3.4) |
|  |  | Significance of Fixed Effects (P value) | | | | | | | | | | | | | | | | | |
| Ecology (E) | | 0.197 | | 0.074 | | 0.876 | | 0.286 | | 1.000 | | 0.228 | | 1.000 | | 0.909 | | 0.507 | |
| Till (T) | | 0.127 | | 0.692 | | 0.876 | | 0.106 | | 1.000 | | 0.774 | | 1.000 | | 0.199 | | 1.000 | |
| E*T | | 1.000 | | 1.000 | | 1.000 | | 0.016 | | 1.000 | | 1.000 | | 1.000 | | 0.710 | | 0.507 | |

Supplemental Figure 1. Depiction of the 13 sampling points evenly distributed along a W-shaped transect adapted to the size and shape of each field for the seedbank sampling.


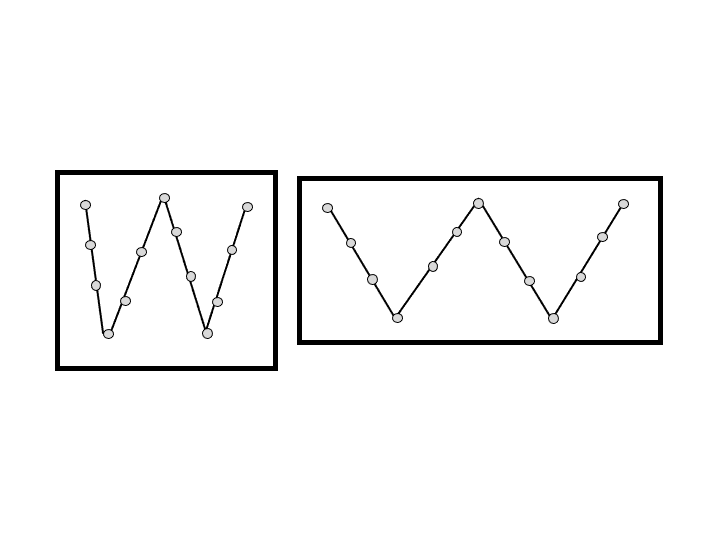


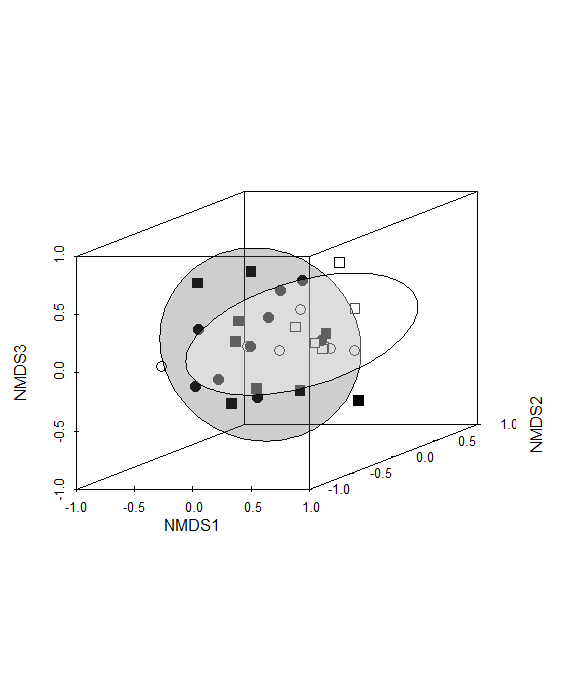


Supplemental Figure 2. Plot scores for the three dimensional explanatory axes of NMDS analysis of weed species composition within the soil seedbank of both conventional (circle) and zero-till (square) farmers' fields in the Upland (open) and Lowland (black) topography in the SVM region. NMDS: stress = 0.16, P < 0·05, cumulative R^2^ for axes 1, 2, and 3 = 0.75.
